# Supplementary material for: Genomic profiling and pre-clinical modelling of breast cancer leptomeningeal metastasis reveals acquisition of a lobular-like phenotype
Source: Nat Commun. 2023 Nov 16;14:7408. doi: 10.1038/s41467-023-43242-x (PMC10654396; doi:10.1038/s41467-023-43242-x)
Supplement: Supplementary file 1 — Supplementary Information [file 41467_2023_43242_MOESM1_ESM.pdf]

Genomic profiling and pre-clinical modelling of breast cancer leptomeningeal metastasis reveals acquisition of a lobular-like phenotype

Amanda Fitzpatrick, Marjan Iravani, Adam Mills, David Vicente, Thanussuyah Alaguthurai, Ioannis Roxanis, Nicholas C. Turner, Syed Haider, Andrew N.J. Tutt and Clare M. Isacke

Supplementary Information

Supplementary Table 1 | Clinicopathological characteristics of patients in BCLM cohort

Supplementary Table 2 | Whole exome sequencing and variant calling metrics

Supplementary Table 3 | Characteristics of BCLM genomic evolution

Supplementary Table 4 | Frequently altered cancer driver genes in plasma cfDNA

Supplementary Table 5 | OncoKB actionable gene alterations in CSF cfDNA

Supplementary Table 6 | Immunohistochemical scoring of primary tumours and matched BCLM PDOs

Supplementary Table 7 | STR authentication of cell lines and PDOs

Supplementary Table 8 | Antibodies used for immunohistochemistry

Supplementary Figure 1 | Metrics of samples undergoing WES

Supplementary Figures 2-5 | Trinucleotide context of single nucleotide substitutions in CSF cfDNA, plasma cfDNA, primary tumour, metastasis.

Supplementary Figure 6 | Correlation of CSF cfDNA unique variants with clinical history

Supplementary Figures 7-13 | BCLM clonal evolution modelling

Supplementary Figure 14 | Cancer-associated mutation and copy number landscape of all matched samples

Supplementary Figure 15 | Comparison of CNA frequency between sample type

Supplementary Figure 16 | BCLM-enriched genomic alterations in biological processes

Supplementary Figure 17 | Comparison of genomic findings in BCLM PDOs and matched primary tumour, plasma cfDNA or metastatic tissue samples

Supplementary Figure 18 | Transduction of BCLM PDOs to express mCherry and luciferase

Supplementary Data 1 | Somatic variants identified by WES (excluding synonymous SNVs)

Supplementary Data 2 | Chemotherapy-related SBS mutational signatures

Supplementary Data 3 | SBS mutational signatures across sample types

Supplementary Data 4 | Genomic alterations in BCLM *vs*. MBC cohort

Supplementary Data 5 | Copy number alterations (standardised log2 ratio) determined by CNVkit analysis of WES data

Supplementary Table 1 | Clinicopathological characteristics of patients in BCLM cohort

	**Patients (*n* = 21)**	
	n	%
**Age (years)**		
Median	52	
Range	28 - 73	
**Time from pBC (years)**		
Median	4.4	
Range	0.70 - 14.7	
**Time from mBC (years)**		
Median	1.1	
Range	0 - 6.5	
**Gender**		
Female	21	100
Male	0	0
**Histological subtype**		
Lobular	11	52.39
Mixed lobular/NST	1	4.76
NST	7	33.33
Micropapillary	1	4.76
Apocrine	1	4.76
**Receptor subtype (primary tumour)**		
ER+ HER2-	13	61.90
ER+ HER2+	2	9.52
ER- HER2+	1	4.76
TN	5	23.81
**Staging at diagnosis**		
N0	5	23.81
N1+	9	42.86
unknown N	1	4.76
M1	6	28.57
**Chemotherapy (adjuvant/neoadjuvant)**		
Yes	14	66.67
No	1	4.76
N/A (M1)	6	28.57
**Endocrine therapy (adjuvant/neoadjuvant)**		
Tamoxifen	3	20.00
Tamoxifen then AI	3	20.00
AI	4	26.67
N/A (M1)	5	33.33
**Other metastatic sites**		
None (BCLM only)	3	14.29
Brain parenchyma	7	33.33
Synchronous	2	9.52
Metachronous	5	23.81
Bone	15	71.43
Serosal	7	33.33
Soft tissue	7	33.33
Liver	4	19.05
Ovary	3	14.29
Nodal	3	14.29
Adrenal	1	4.76
Lung	1	4.76

AI, aromatase inhibitor; BCLM,breast cancer leptomeningeal metastasis; ER, oestrogen receptor; HER2, human epidermal growth factor receptor 2; M1, metastatic stage 1; mBC, metastatic breast cancer; N, nodal status; N/A, not applicable; pBC, primary breast cancer; TN, triple negative.

Supplementary Table 2 | Whole exome sequencing and variant calling metrics

	**Mean coverage (Picard)**	**Somatic variants (total)**	**SNV (total)**	**Indel (total)**
	Median (IQR)	*Median (Range)*	*Median (Range)*	*Median (Range)*
**CSF cfDNA**	164.7 (104.8 - 211)	125 (40 - 1196)	112 (36 - 782)	9 (4 - 560)
**Plasma cfDNA**	316.9 (283.7 - 362.3)	124 (34 - 464)	116 (30 - 379)	7 (3 - 116)
**Primary tumour**	195.6 (131.9 - 236.4)	80 (43 - 700)	76 (42 - 373)	7 (1 - 327)
**Metastasis**	211.3 (178.3 - 233.9)	76 (46 - 418)	70 (43 - 279)	5 (3 - 139)
**Germline**	106 (100.1 - 117.8)	- (-)	- (-)	- (-)

CSF, cerebrospinal fluid; IQR,interquartile range; SNV,single nucleotide variation.

Supplementary Table 3 | Characteristics of BCLM genomic evolution

Study ID	CSF seeding from primary tumour	CSF and plasma evolution	Metastatic seeding pattern
**KCL566**	early	divergent	monophyletic
**RMH010**	early	divergent	monophyletic
**KCL590**	early	divergent	monophyletic
**KCL523**	early	divergent	monophyletic
**KCL610**	early	divergent	monophyletic
**KCL499**	early	divergent	monophyletic
**KCL448**	early	divergent	monophyletic
**KCL622**	early	linear	monophyletic
**KCL553**	early	N/A	polyphyletic
**KCL320**	early	N/A	N/A
**KCL617**	early	N/A	N/A
**KCL449**	early	N/A	N/A
**KCL148**	early	N/A	N/A
**KCL450**	early	N/A	N/A
**KCL616**	early	N/A	N/A
**KCL680**	early	N/A	N/A
**RMH008**	N/A	divergent	N/A
**KCL658**	N/A	divergent	N/A
**KCL650**	N/A	linear	N/A
**RMH011**	N/A	N/A	N/A

N/A, not assessable

Supplementary Table 4 | Frequently altered cancer driver genes in plasma cfDNA

Gene	Altered in plasma	Altered in plasma (unqiue)	Total plasma samples sequenced	% of plasma samples with alteration (total)	% plasma samples with alteration (unique to plasma cfDNA)	Frequently altered in CSF cfDNA?
PER1	5	5	11	45%	45%	no
TP53	5	0	11	45%	0%	yes
SRSF2	4	3	11	36%	27%	no
BRCA2	4	2	11	36%	18%	yes
CDH1	4	0	11	36%	0%	yes
MDM2	4	0	11	36%	0%	yes
NUTM2A	3	3	11	27%	27%	yes
PTPN6	3	3	11	27%	27%	no
STK11	3	3	11	27%	27%	no
FLNA	3	2	11	27%	18%	no
CCND1	3	0	11	27%	0%	yes
HMGA2	3	0	11	27%	0%	no
MUC16	3	0	11	27%	0%	yes
PIK3CA	3	0	11	27%	0%	yes
PTEN	3	0	11	27%	0%	yes
PTPRB	3	0	11	27%	0%	yes
CALR	2	2	11	18%	18%	no
DNAJB1	2	2	11	18%	18%	no
LYL1	2	2	11	18%	18%	no
MUC4	2	2	11	18%	18%	no
BAZ1A	2	1	11	18%	9%	no
CLP1	2	1	11	18%	9%	no
ERBB2	2	1	11	18%	9%	yes
FAT1	2	1	11	18%	9%	no
MYH11	2	1	11	18%	9%	no
RB1	2	1	11	18%	9%	yes
RUNX1	2	1	11	18%	9%	yes
TFPT	2	1	11	18%	9%	no
ATM	2	0	11	18%	0%	yes
BCL11B	2	0	11	18%	0%	no
DROSHA	2	0	11	18%	0%	no
FAT4	2	0	11	18%	0%	no
LRP1B	2	0	11	18%	0%	no
MYCN	2	0	11	18%	0%	no
ZNF429	2	0	11	18%	0%	no

Supplementary Table 5 | OncoKB actionable gene alterations in CSF cfDNA

Patient ID	Gene	Alteration type	Alteration detail	Therapeutic level (any cancer)	Therapeutic level (breast cancer)	Predicts/may predict repsonse to*	Shared with primary tumour
KCL148	*BRCA1*	Frameshift insertion	p.Y655Vfs*18	1	3A	Olaparib	not shared
KCL566	N/A	High TMB	TMB >10 muts/Mb	1	1	Pembrolizumab	not shared
KCL566	*NF1*	Frameshift deletion	p.Y1586Ifs*17	4	4	Trametinib; Cobimetinib	not shared
KCL610	*BRCA2*	Frameshift deletion	p.N1599Ifs*18	1	3A	Olaparib	not shared
KCL680	*ESR1*	Nonsynonymous SNV	p.Y537S	1	1	Fulvestrant	not shared
KCL148	N/A	High TMB	TMB >10 muts/Mb	1	1	Pembrolizumab	shared
KCL148	*ARID1A*	Frameshift deletion	p.P1468Lfs*13	4	4	Tazemetostat	shared
KCL148	*PTEN*	Frameshift insertion	p.E157Gfs*23	4	4	GSK2636771	shared
KCL148	*PTEN*	Frameshift insertion	p.G251Vfs*6	4	4	GSK2636771	shared
KCL148	*PTCH1*	Frameshift insertion	p.R1242Qfs*17	3B	none	Sonidegib,Vismodegib	shared
KCL320	*PIK3CA*	Nonsynonymous SNV	p.E542K	1	1	Alpelisib	shared
KCL448	*CDKN2A*	Stopgain	p.R80X	4	4	Abemaciclib	shared
KCL449	*PIK3CA*	Nonsynonymous SNV	p.H1047R	1	1	Alpelisib	shared
KCL450	*AKT1*	Nonsynonymous SNV	p.E17K	3A	3A	Capivasertib	shared
KCL523	*BRCA2*	Nonsynonymous SNV	p.R3052W	1	3A	Olaparib	shared
KCL523	*PIK3CA*	Nonsynonymous SNV	p.N1044K	2	2	Alpelisib	shared
KCL553	*ARID1A*	Frameshift deletion	p.Q548Hfs*65	4	4	Tazemetostat	shared
KCL566	*MDM2*	Amplification	Amplification	4	none	Milademetan	shared
KCL590	*MDM2*	Amplification	Amplification	4	none	Milademetan	shared
KCL610	*PTEN*	Frameshift deletion	p.F21Yfs*16	4	4	GSK2636771	shared
KCL610	*MDM2*	Amplification	Amplification	4	none	Milademetan	shared
KCL616	*PIK3CA*	Nonsynonymous SNV	p.H1047R	1	1	Alpelisib	shared
KCL616	*ARID1A*	Stopgain	p.Q920X	4	4	Tazemetostat	shared
KCL617	*ERBB2*	Amplification	Amplification	1	1	Trastuzumab	shared
KCL622	*ATM*	Nonsynonymous SNV	p.R337H	1	none	Olaparib	shared
KCL650	*ATM*	Splice site	Splice variant	1	none	Olaparib	shared
RMH008	*PIK3CA*	Nonsynonymous SNV	p.E39K	2	2	Alpelisib	shared
RMH008	*ERBB2*	Nonsynonymous SNV	p.L755S	2	3A	Neratinib	shared
RMH011	*ERBB2*	Amplification	Amplification	1	1	Trastuzumab	shared

*prediction of drug response not know for CNS metastasis; TMB, tumour mutational burden

Supplementary Table 6 | IHC scoring of primary tumours and matched BCLM PDOs

Study ID	Primary tumour	BCLM PDO
KCL320	ER 8; HER2 0	ER 0; HER2 3+
KCL450	ER 8; HER2 0	ER 0; HER2 1+
KCL566	ER 6; HER2 0	ER 3; HER2 2+
KCL622	ER 0; HER2 0	ER 0; HER2 0
KCL625	ER 3; HER2 0	ER 0; HER2 1+

Scoring performed by independent pathologist (I.R.)

Supplementary Table 7 | STR authentication of cell lines and PDOs

Cell line/PDO ID	Last date authentication performed
DU4475	30/03/2021
MDA-MB-134-VI	19/01/2021
T47D	19/01/2021
SUM44PE	19/01/2021
MCF7$^{CDH1+/+}$	09/03/2017
MCF7$^{CDH1-/-}$	09/03/2017
KCL320	19/01/2021
KCL450	30/03/2021
KCL566	30/03/2021
KCL622	30/03/2021
KCL645	30/03/2021

The results for all cell lines used agreed with the public databases, confirming the correct identity of each cell line used. The results for PDOs matched the original authentication and confirming the correct identity.

Supplementary Table 8 | Antibodies used for immunohistochemistry.

Antibody	Species	Source (Catalogue no.)	Dilution
Alpha-catenin	Rabbit	Abcam (51032)	1:100
Beta-catenin	Mouse	BD Biosciences (610153)	1:2500
E-cadherin	Mouse	Dako (M3612)	1:50
ERα	Rabbit	Dako (M3643)	1:80
HER2	N/A	Dako (SK001)	N/A
Lamin A/C	Rabbit	Abcam (108595)	1:750
p120-catenin	Mouse	BD Biosciences (610134)	1:500

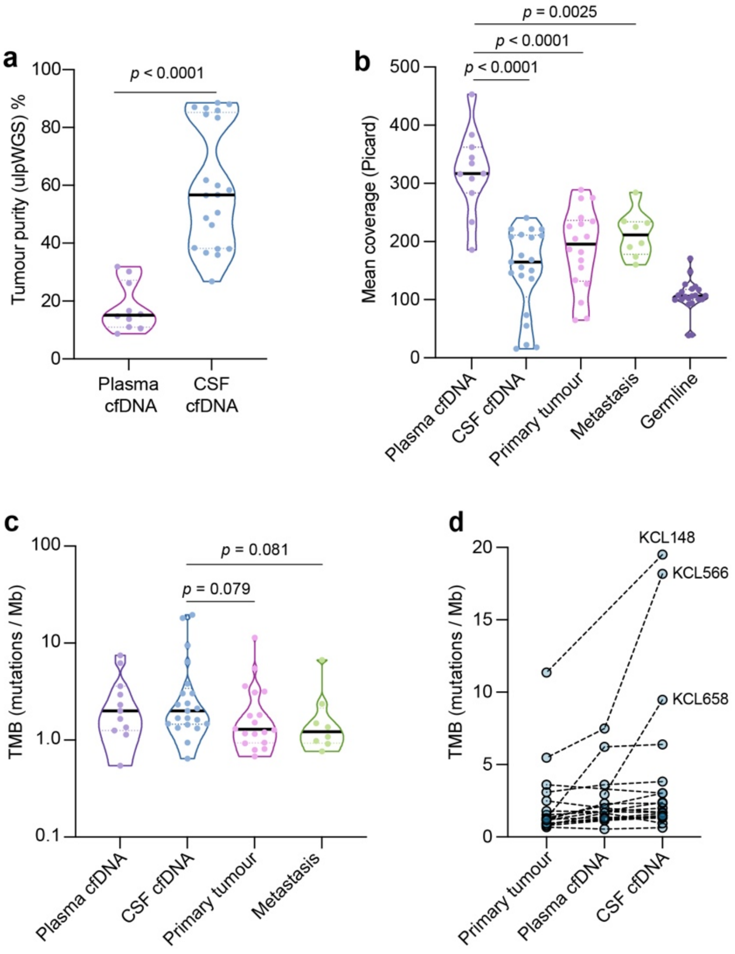

Supplementary Fig. 1. Metrics of samples undergoing WES**. a** Tumour purity of cfDNA assessed by ulpWGS (ultra-low-pass whole genome sequencing) within the concurrently collected CSF (n = 21) and plasma (n = 11) samples. The 10 plasma cfDNA samples which did not undergo WES (due to tumour content <10%) are not shown. Significance by Mann-Whitney test, two-tailed. **b** Sequencing coverage of all samples. Plasma cfDNA, n = 11; CSF cfDNA, n = 21; primary tumour, n = 18; metastasis, n = 8; germline, n = 21. Significance by Mann-Whitney test, two-tailed. **c** Tumour mutational burden (TMB) in samples shown in panel b. Significance by Mann-Whitney test, two-tailed, p-values <0.1 are shown. **d** Matched comparison of TMB in primary tumour, plasma cfDNA and CSF cfDNA samples collected from individual patients. Source data for all panels are provided as a Source Data file.

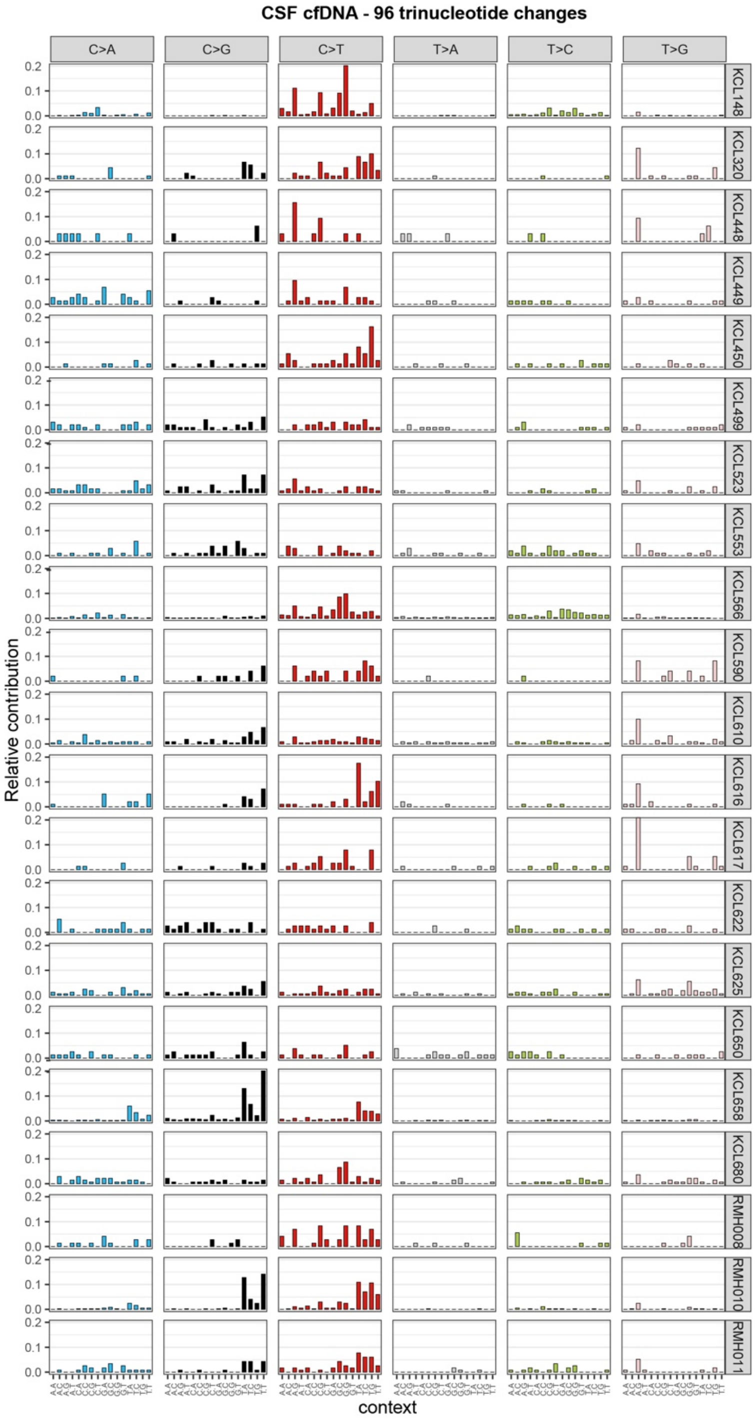

Supplementary Fig. 2. Trinucleotide context of single nucleotide substitutions in CSF cfDNA. Data associated with Figure 1c and Supplementary Data 2 and 3. The x-axis indicates the 5′ and 3′ nucleotides for each of the top panel substitutions for the three base-pair motifs. The y-axis shows the single-base substitution (SBS) composition of each sample by the 96 trinucleotide sequence motifs, grouped by sample type.

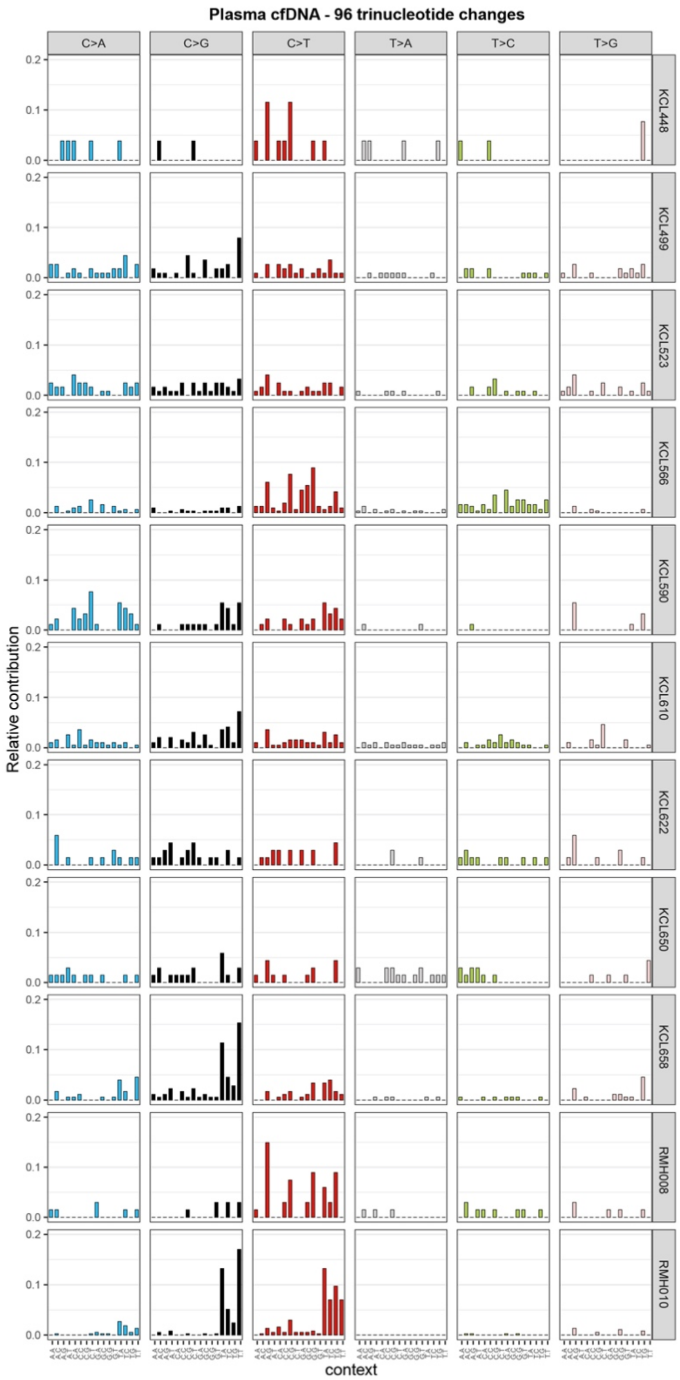

Supplementary Fig. 3. Trinucleotide context of single nucleotide substitutions in plasma cfDNA. Data associated with Figure 1c and Supplementary Data 2 and 3. The x-axis indicates the 5′ and 3′ nucleotides for each of the top panel substitutions for the three base-pair motifs. The y-axis shows the single-base substitution (SBS) composition of each sample by the 96 trinucleotide sequence motifs, grouped by sample type.

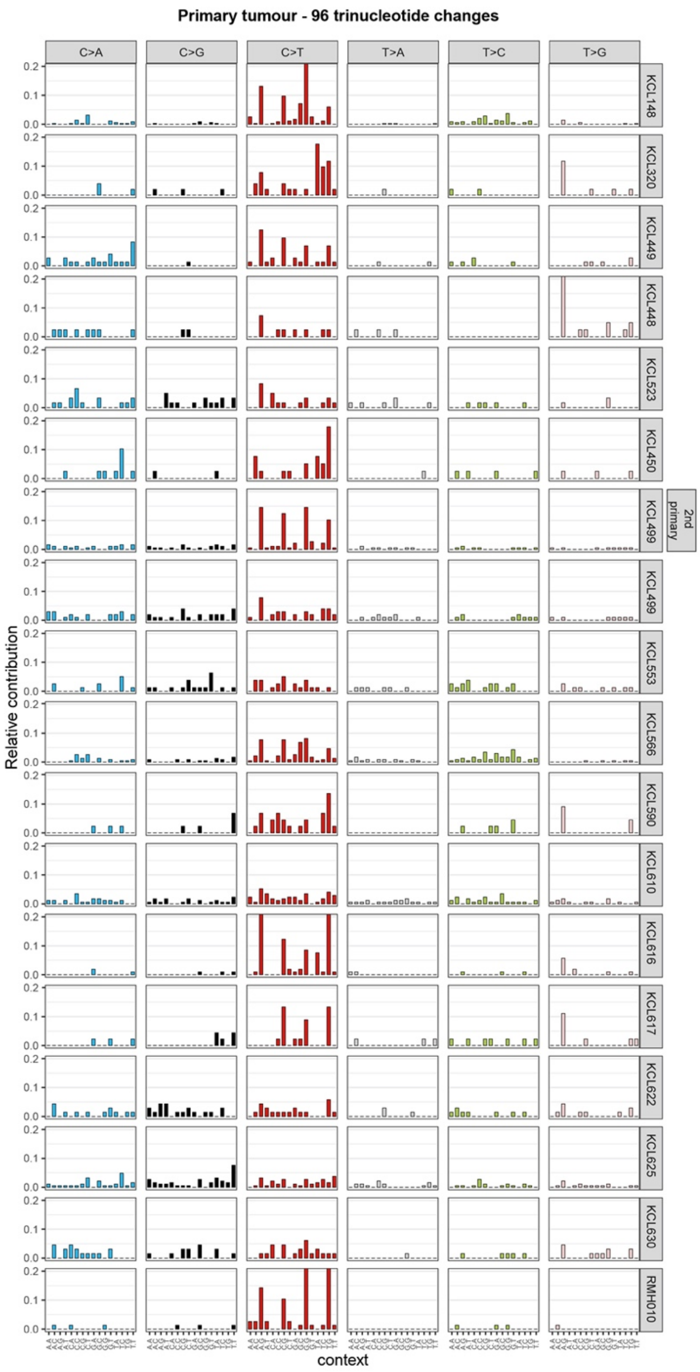

Supplementary Fig. 4. Trinucleotide context of single nucleotide substitutions in primary tumours. Data associated with Figure 1c and Supplementary Data 2 and 3. The x-axis indicates the 5′ and 3′ nucleotides for each of the top panel substitutions for the three base-pair motifs. The y-axis shows the single-base substitution (SBS) composition of each sample by the 96 trinucleotide sequence motifs, grouped by sample type.

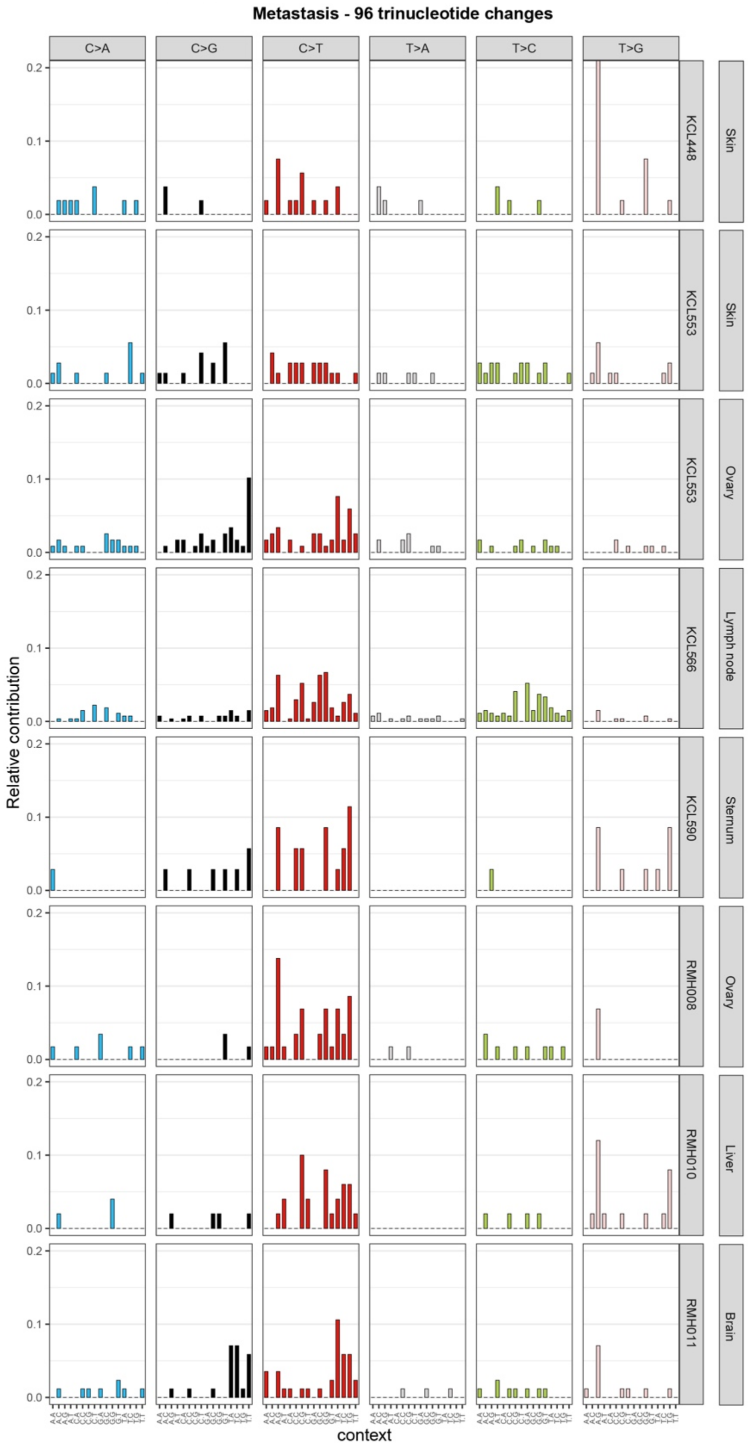

Supplementary Fig. 5. Trinucleotide context of single nucleotide substitutions in metastatic samples. Data associated with Figure 1c and Supplementary Data 2 and 3. The x-axis indicates the 5′ and 3′ nucleotides for each of the top panel substitutions for the three base-pair motifs. The y-axis shows the single-base substitution (SBS) composition of each sample by the 96 trinucleotide sequence motifs, grouped by sample type.

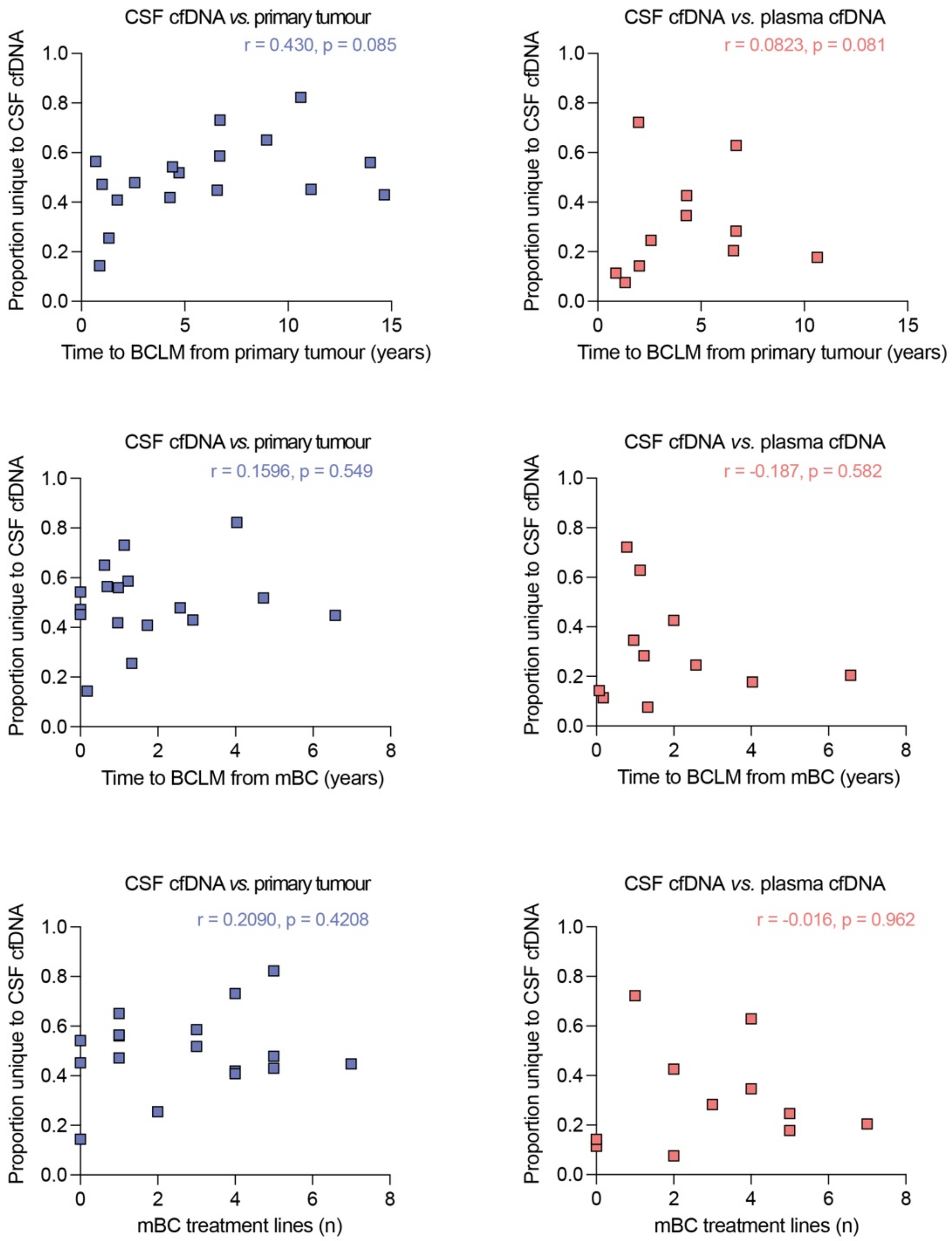

Supplementary Fig. 6. Correlation of CSF cfDNA unique variants with clinical history**.** Correlation of proportion of variants unique to CSF cfDNA *vs*. primary tumour (left panels) (*n* = 17 pairs) and CSF cfDNA *vs.* matched plasma cfDNA (*n* = 11 pairs) (right panels) with time to BCLM diagnosis from primary tumour diagnosis, time to BCLM diagnosis time from diagnosis of metastatic breast cancer, and to number of previous systemic therapies (treatment lines) completed. Pearson's r correlation, two-tailed p-values. Source data are provided as a Source Data file.

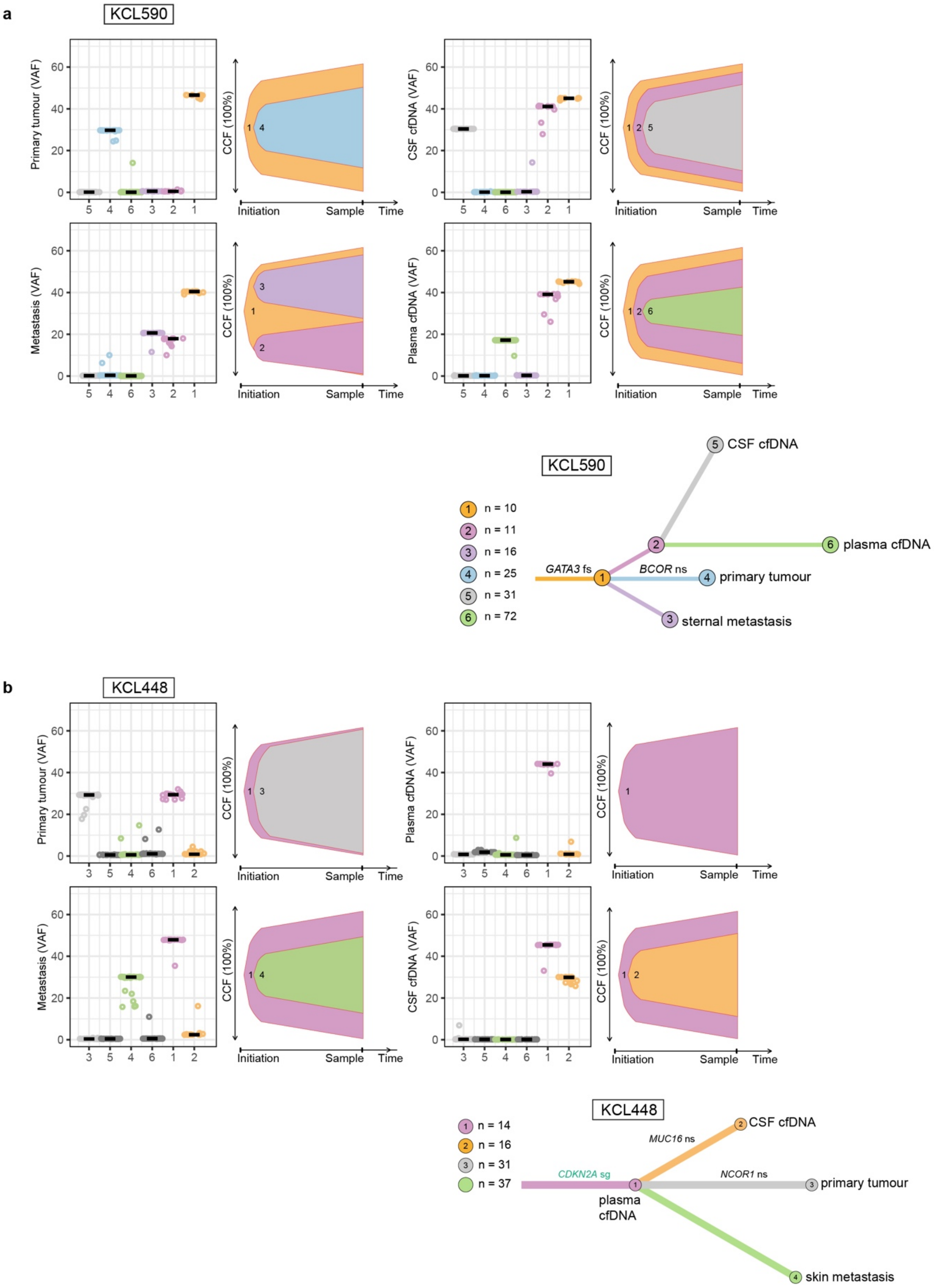

Supplementary Fig. 7. BCLM clonal evolution modelling. Data associated with Figure 2.

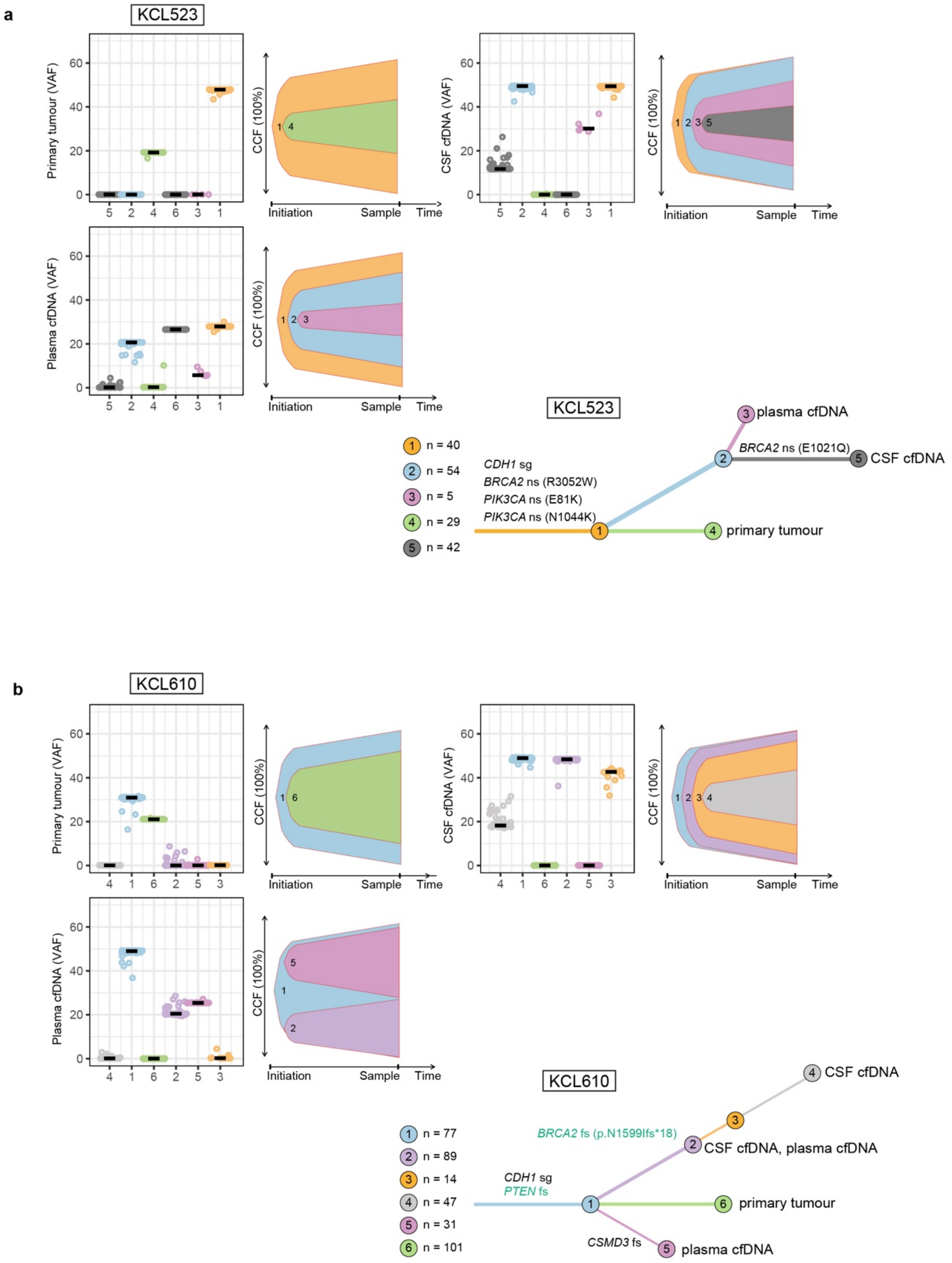

Supplementary Fig. 8. BCLM clonal evolution modelling. Data associated with Figure 2.

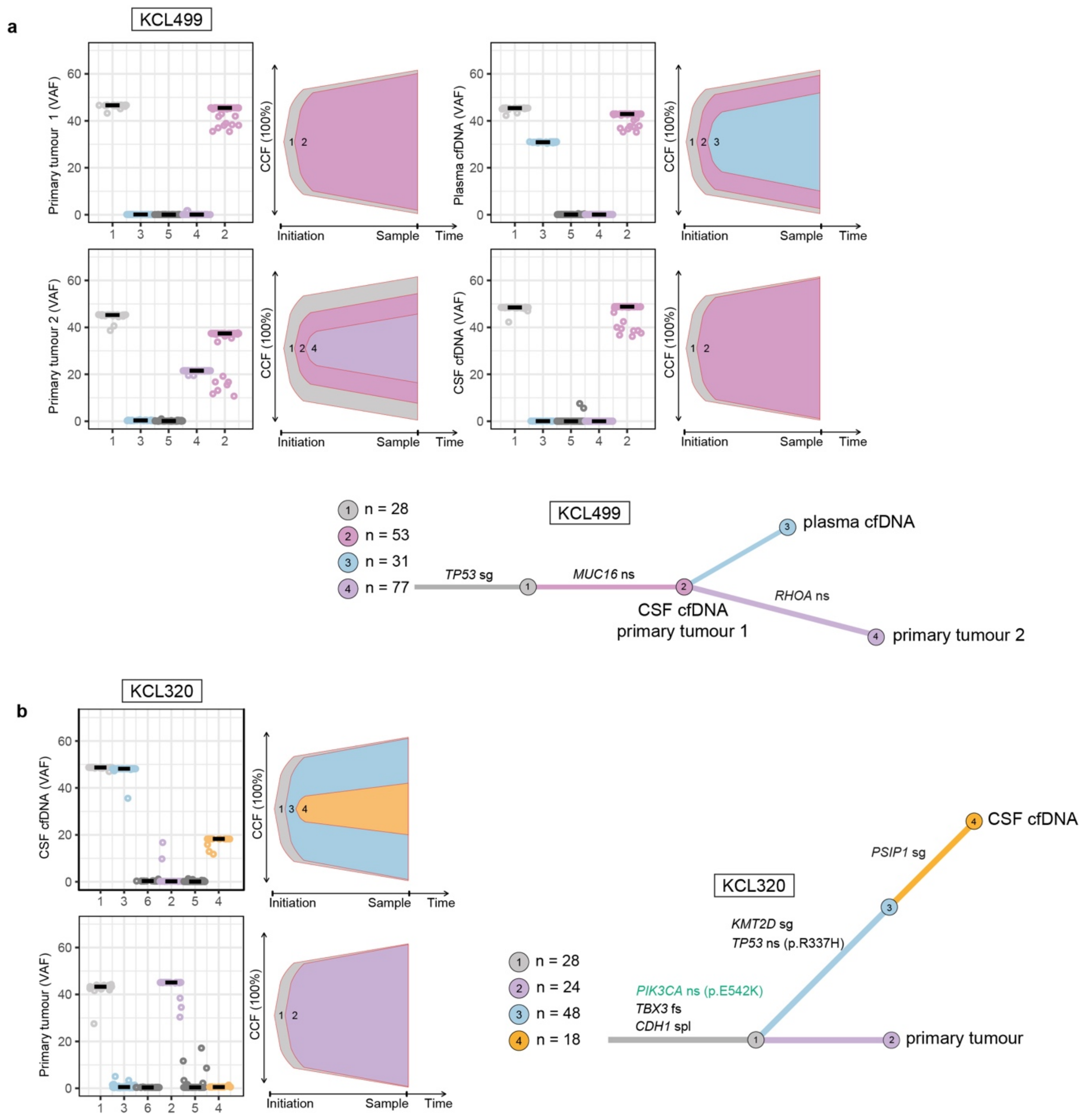

Supplementary Fig. 9. BCLM clonal evolution modelling. Data associated with Figure 2.

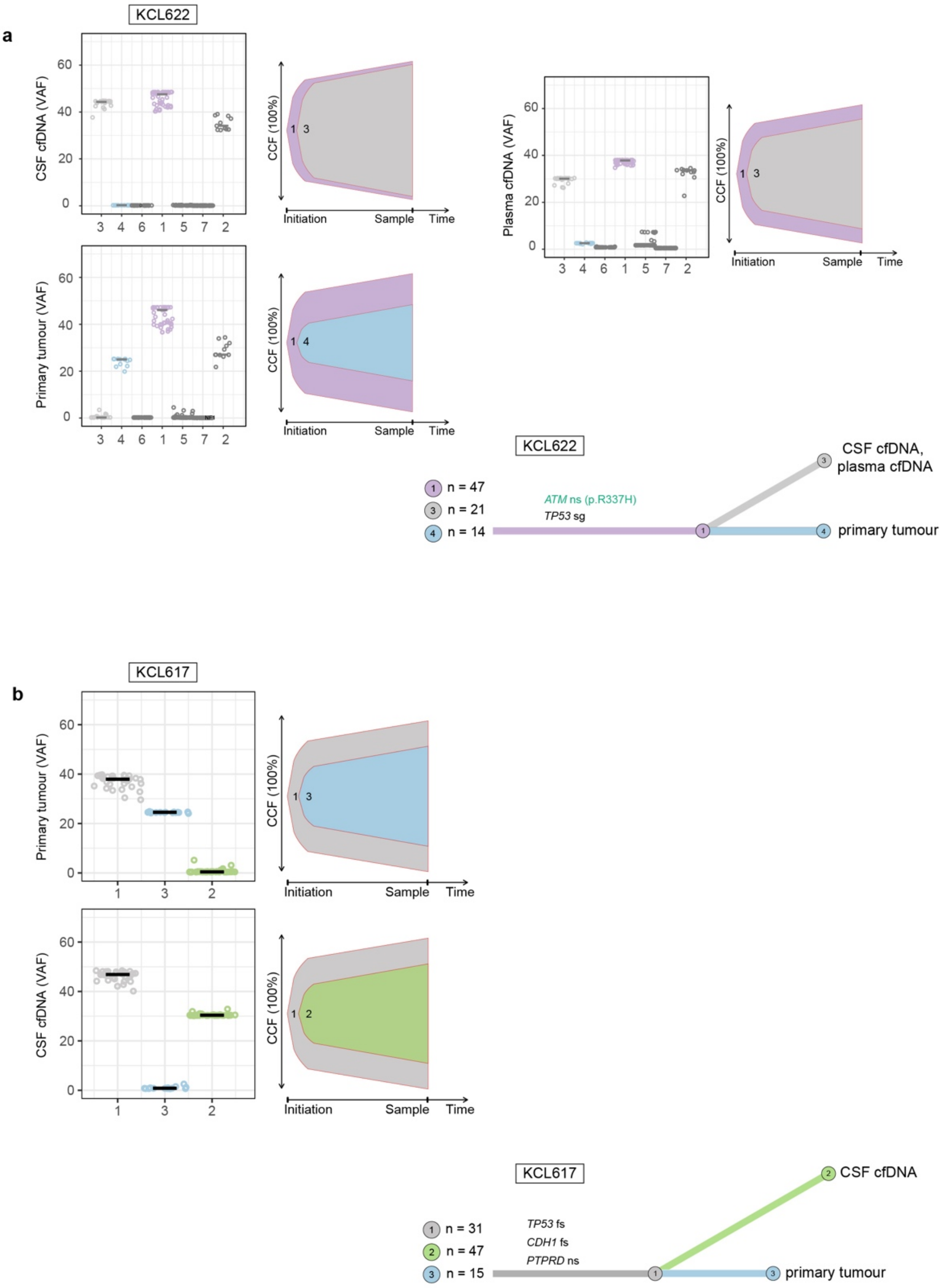

Supplementary Fig. 10. BCLM clonal evolution modelling. Data associated with Figure 2.

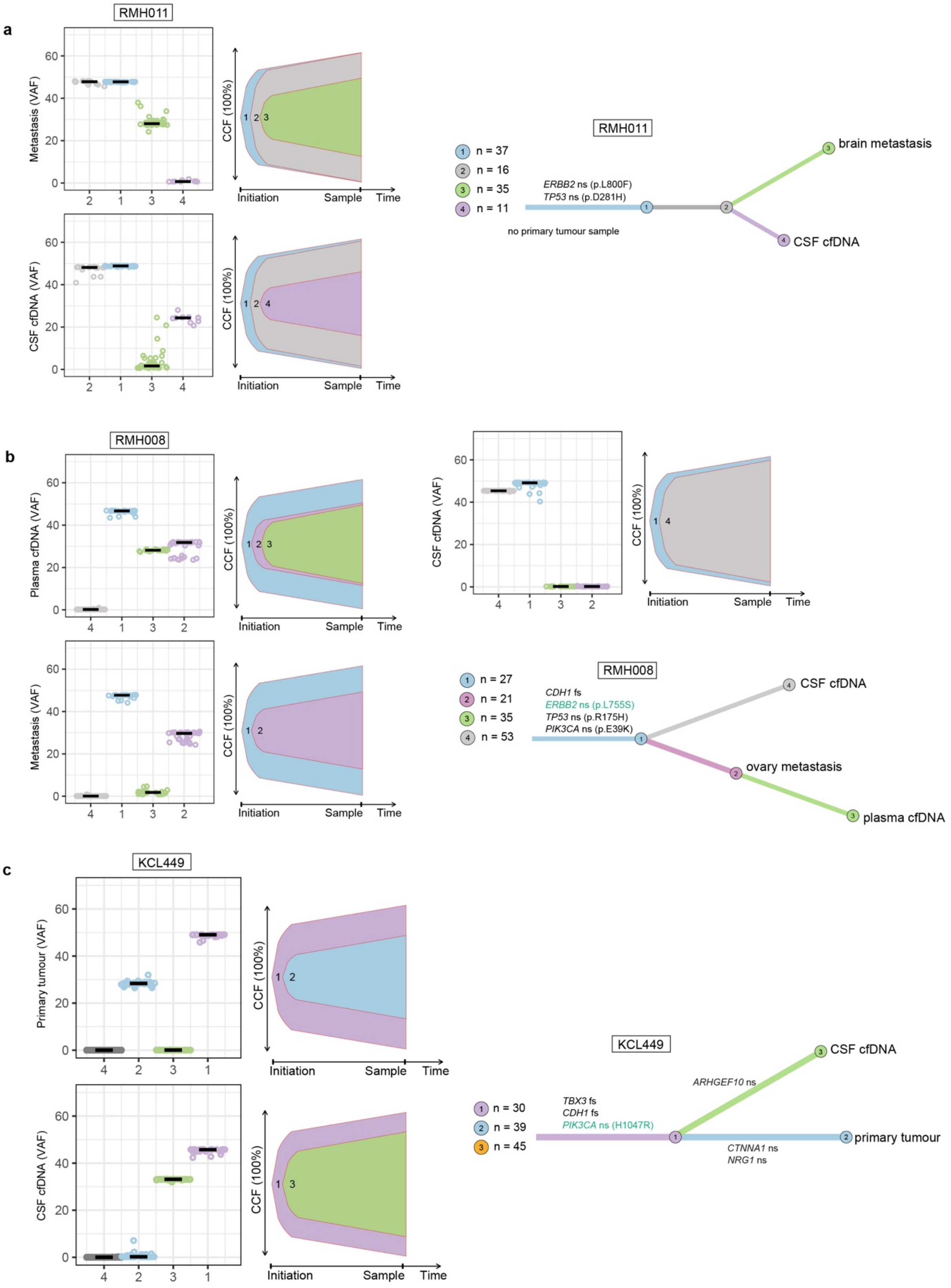

Supplementary Fig. 11. BCLM clonal evolution modelling. Data associated with Figure 2.

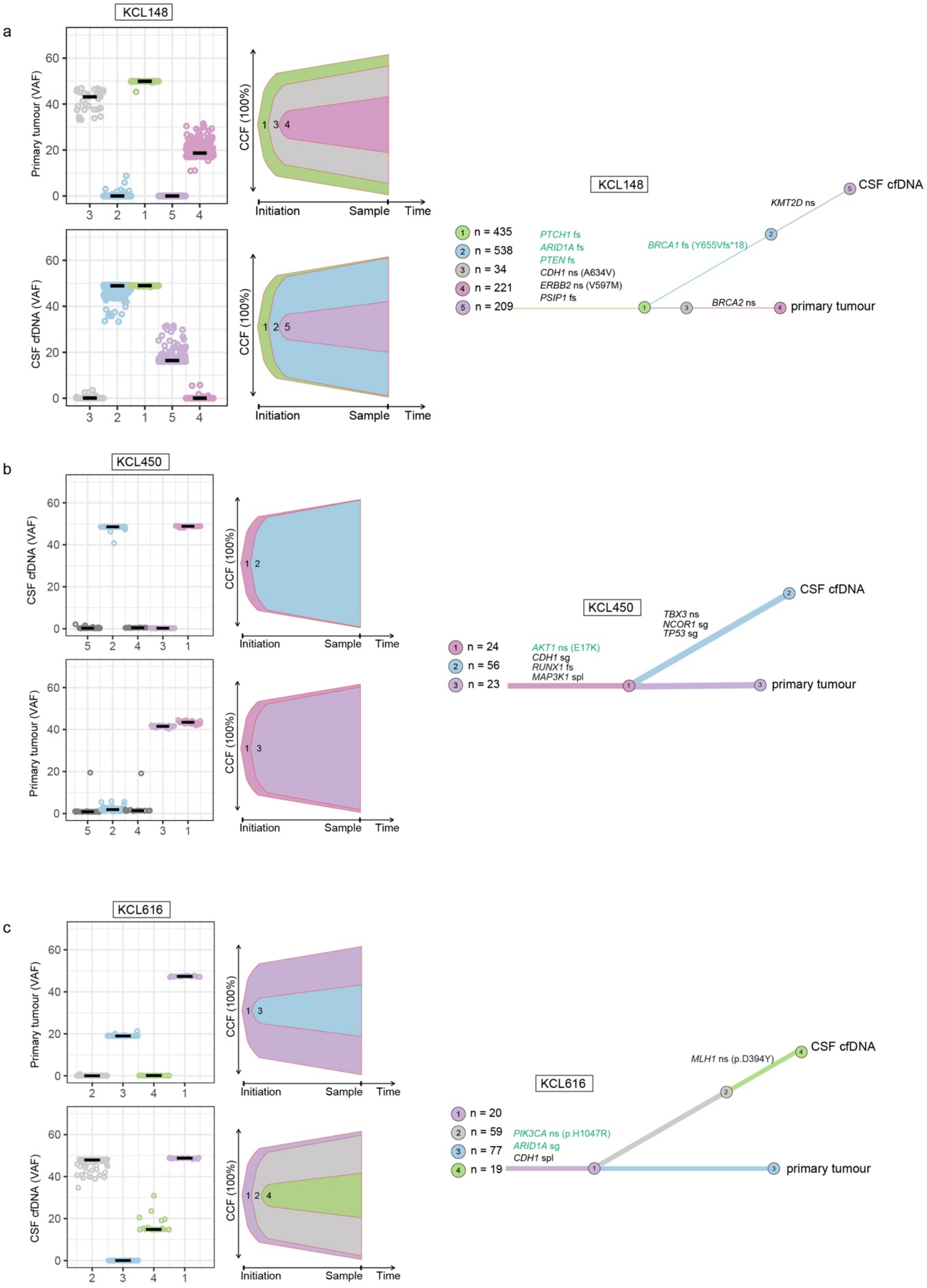

Supplementary Fig. 12. BCLM clonal evolution modelling. Data associated with Figure 2.

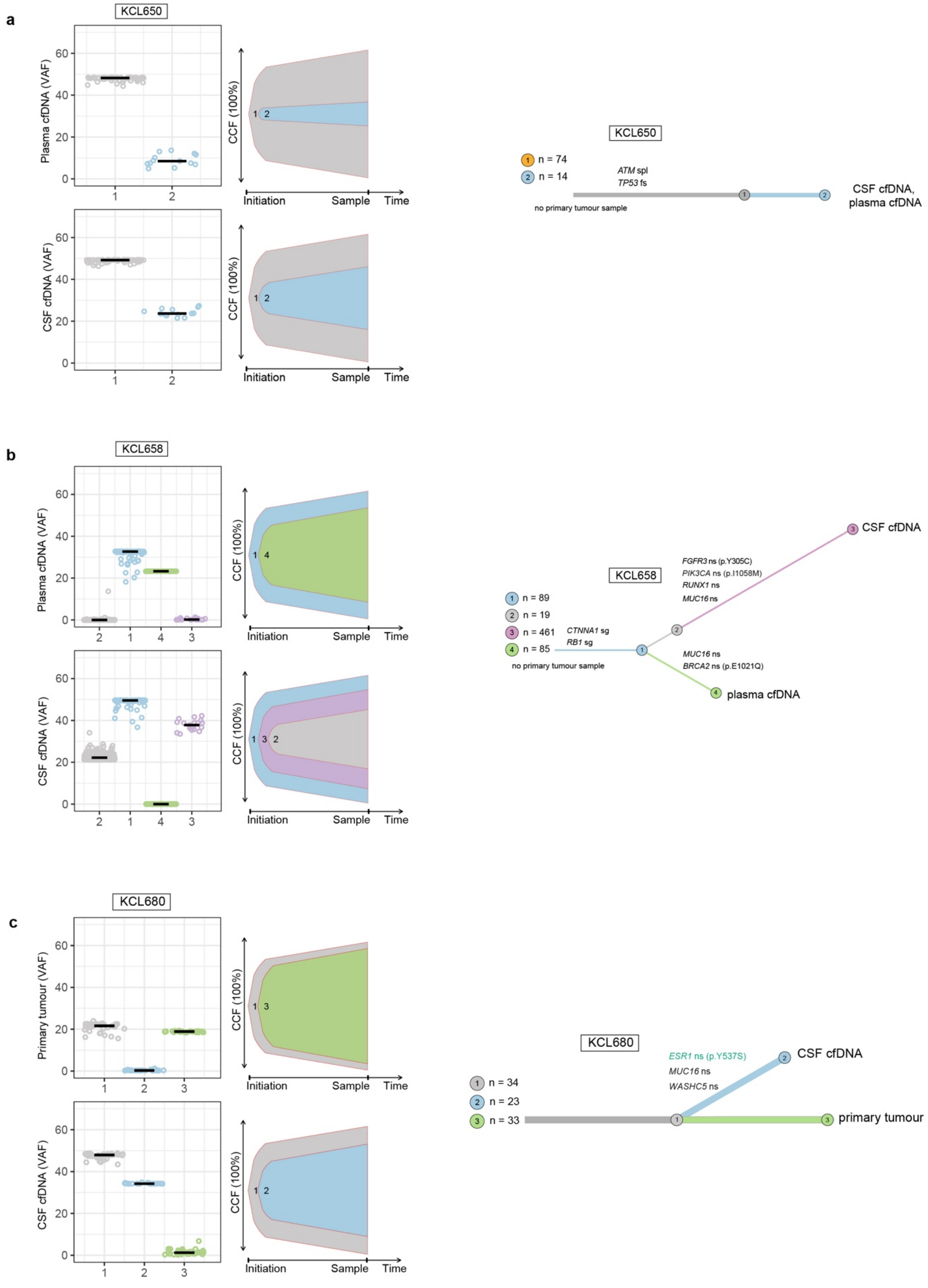

Supplementary Fig. 13. BCLM clonal evolution modelling. Data associated with Figure 2.

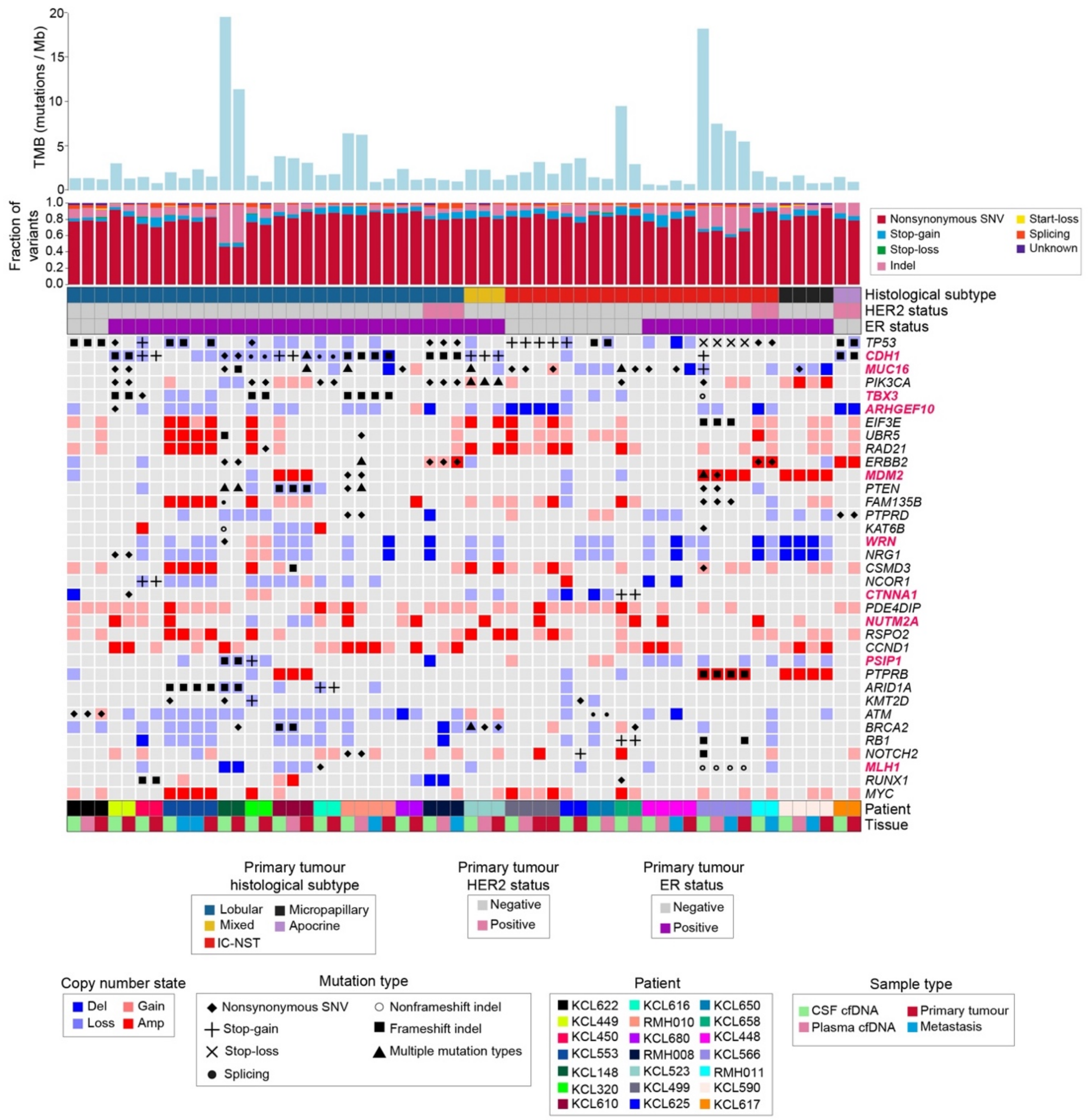

Supplementary Fig. 14. Cancer-associated mutation and copy number landscape of all matched samples. Associated with Fig. 3. Uppermost bar plot show tumour mutational burden (TMB) by sample. Stacked bar chart below shows mutational subtype for each sample as percentage of all variants (excluding synonymous). Banners indicate primary tumour histological subtype and HER2 and ER status. Main plot shows the 36 most frequently cancer-associated altered genes (alterations include mutation and high-level copy number events) in CSF cfDNA as follows; Cancer Gene Census (CGC) gene altered in at least 4 cases; breast cancer driver gene (definition in Methods) altered in at least 3 cases. Gene names are highlighted in red if genomic alteration rate was significantly different ($p \leq 0.05$ by Chi-square test two-sided) in BCLM cohort compared to a publicly available dataset of 216 metastatic breast cancer (MBC cohort) samples (see Methods). Extended data in Supplementary Data 4 displays the significance values for this comparison.

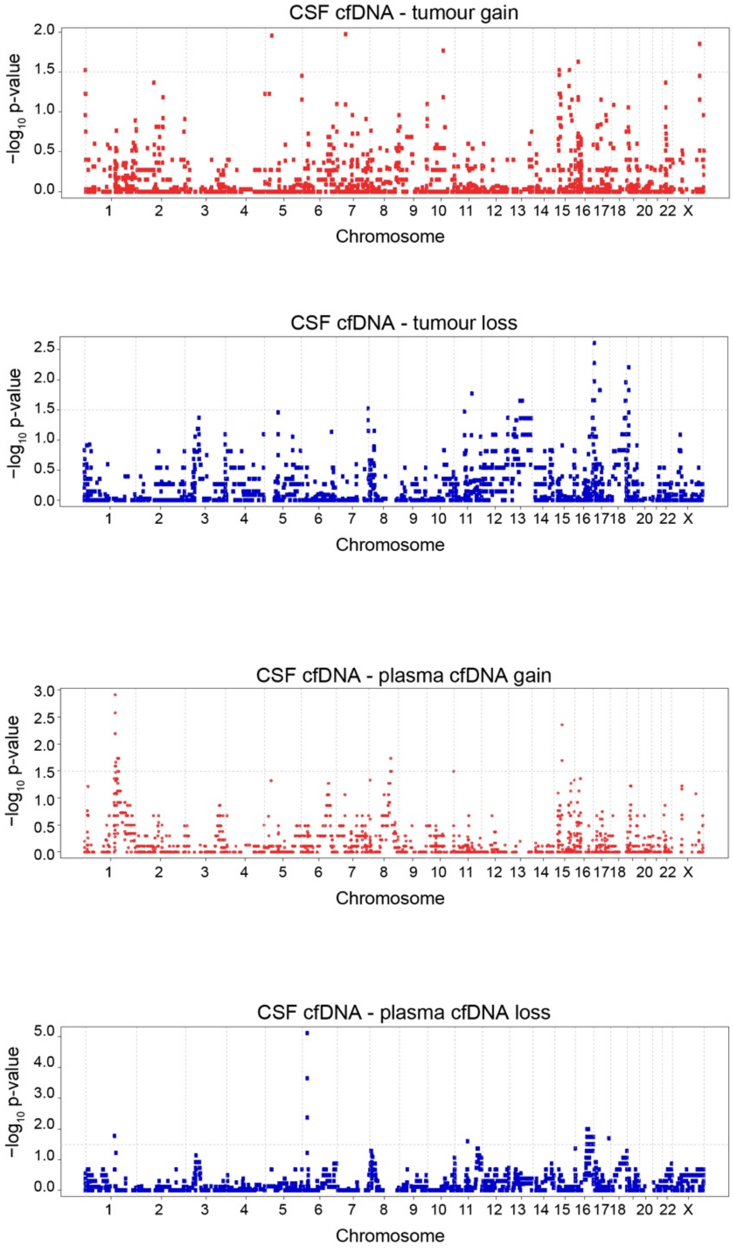

Supplementary Fig. 15. Comparison of CNA frequency between sample type. Associated with Fig. 4a,b. Copy number alteration (CNA) across the genome was determined using WES and adjusted for sample purity and ploidy. Differences in CNA frequency between sample types were analysed with two-sided tests of equal proportions, comparing CSF cfDNA and tumour (*n* = 17 pairs), and CSF cfDNA and plasma cfDNA (*n* = 11). Genome-wide plots show the inverse p-value (-log10 p-value) of the significance of CNA frequency difference, where each datapoint represents a gene.

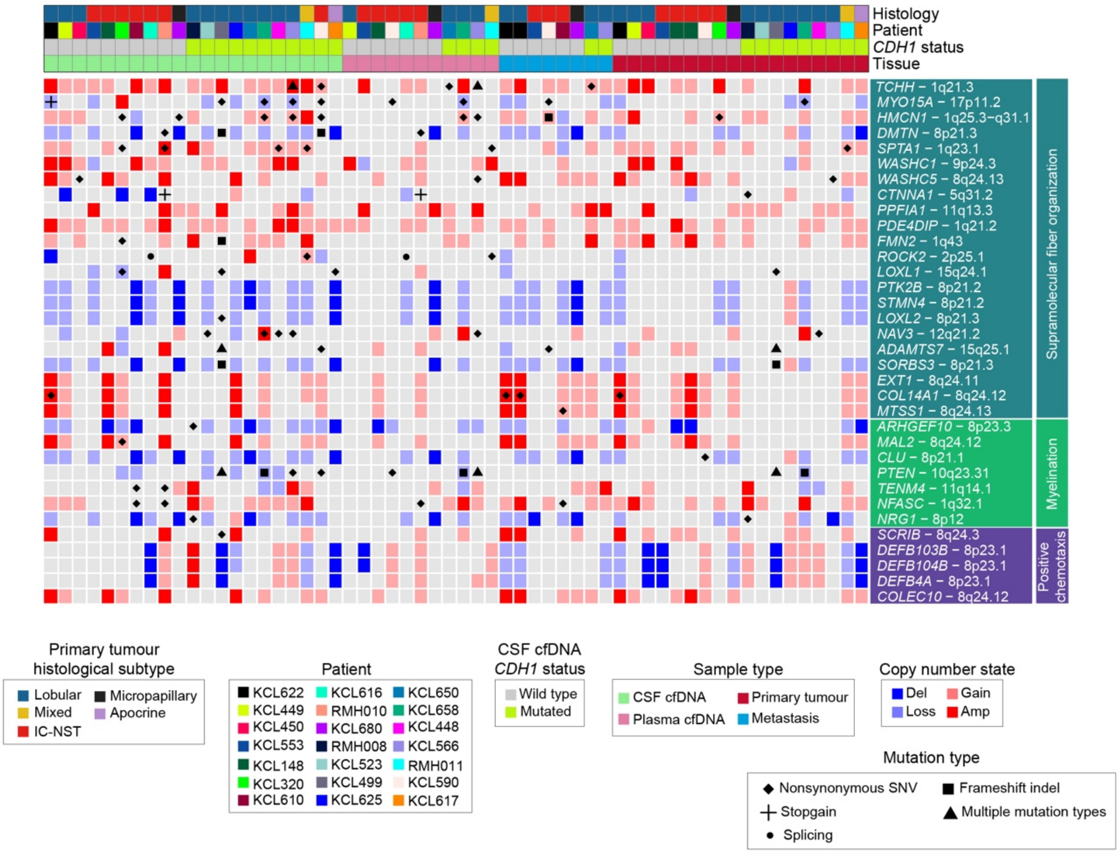

Supplementary Fig. 16. BCLM-enriched genomic alterations in biological processes. Associated with Fig. 4d. Genomic alterations in GO biological processes with -$\log_{10}$ p-value ≥3.0 for enrichment in CSF cfDNA *vs.* primary tumour. Genomic alterations are shown for all sample types in 21 individuals. Banners above indicate; primary tumour histological subtype, patients, *CDH1* mutation status of CSF cfDNA and sample type.

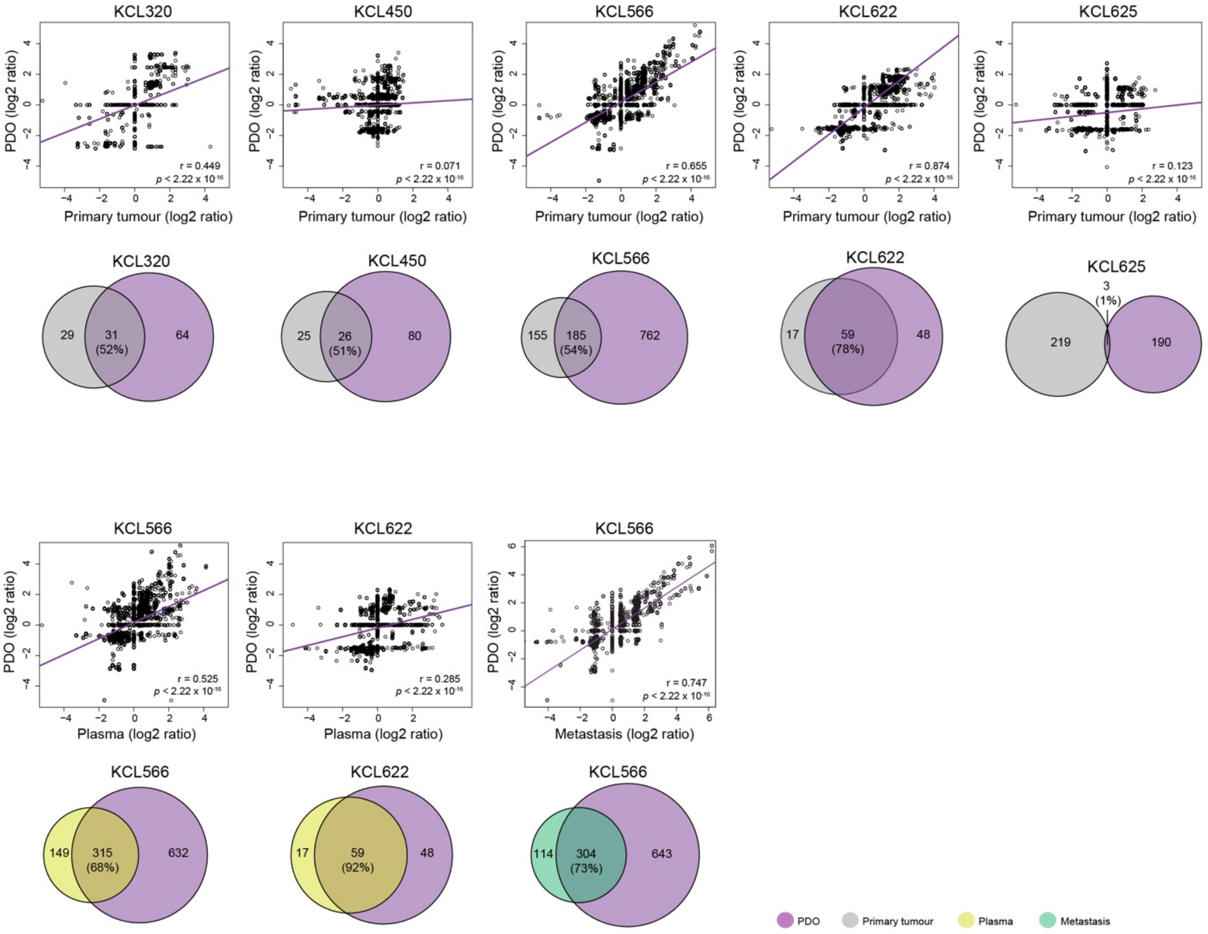

Supplementary Fig. 17. Comparison of genomic findings in BCLM PDOs and matched primary tumour, plasma cfDNA or metastatic tissue samples. Associated with Fig. 6a. Top panel, correlation plots of CNA (standardised log2 ratio, one data point per gene) with regression line plotted (purple) and Pearson's r value plus two-sided significance values are shown. Lower panel, shared variants between primary tumour, metastasis or plasma cfDNA and BCLM PDO. Numbers within circles show mutation count, with the percentage showing variants in sample which also detected in BCLM PDO.

Supplementary Figure 18

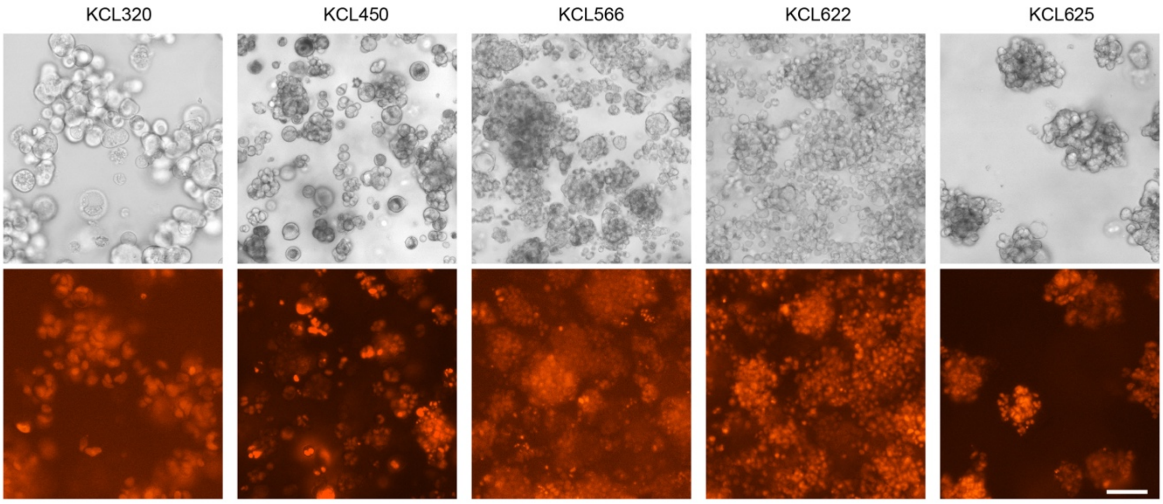

Supplementary Fig. 18. Transduction of BCLM PDOs to express mCherry and luciferase. PDOs were transduced with a lentivirus encoding luciferase and mCherry (mChLuc2) and imaged on an EVOS microscope during 3D culture in bright field and fluorescence mode. Scale bar, 100 µm.
