# Supplementary material for: Genomic profiling and pre-clinical modelling of breast cancer leptomeningeal metastasis reveals acquisition of a lobular-like phenotype
Source: Nat Commun. 2023 Nov 16;14:7408. doi: 10.1038/s41467-023-43242-x (PMC10654396; doi:10.1038/s41467-023-43242-x)
Supplement: Supplementary file 3 — Description of Additional Supplementary Files Document [file 41467_2023_43242_MOESM3_ESM.pdf]

## Description of Additional Supplementary Files

**Supplementary Data 1**

Description: Somatic variants identified by WES (excluding synonymous SNVs). Filtered non-silent somatic variants across all samples are shown

**Supplementary Data 2**

Description: Chemotherapy-related SBS mutational signatures. Chemotherapy-related SBS mutational signature (SBS11, SBS17b, SBS28, SBS31, SBS35, SBS86) analysis across all samples was performed using the WES-identified single nucleotide variants (SNVs) (median of 96 filtered SNV per sample).

**Supplementary Data 3**

Description: SBS mutational signatures across sample types. Mutational signature analysis was performed using the WES-identified single nucleotide variants (SNVs) (median of 96 filtered SNV per sample) providing a mean signature contribution between sample types

**Supplementary Data 4**

Description: Genomic alterations in BCLM vs. MBC cohort. Proportion test (p-value) comparing BCLM altered genes to the MBC cohort (a publicly available dataset of 216 non-BCLM metastatic breast cancer samples).

**Supplementary Data 5**

Description: Copy number alterations (standardised log2 ratio) determined by CNVkit analysis of WES data. Alterations across all samples are shown.
