# Supplementary material for: Genomic profiling and pre-clinical modelling of breast cancer leptomeningeal metastasis reveals acquisition of a lobular-like phenotype
Source: Nat Commun. 2023 Nov 16;14:7408. doi: 10.1038/s41467-023-43242-x (PMC10654396; doi:10.1038/s41467-023-43242-x)
Supplement: Supplementary file 5 — Reporting Summary [file 41467_2023_43242_MOESM5_ESM.pdf]

nature portfolio

Corresponding author(s): Clare M Isacke

Last updated by author(s): YYYY-MM-DD

Reporting Summary

Nature Portfolio wishes to improve the reproducibility of the work that we publish. This form provides structure for consistency and transparency in reporting. For further information on Nature Portfolio policies, see our Editorial Policies and the Editorial Policy Checklist.

Statistics

For all statistical analyses, confirm that the following items are present in the figure legend, table legend, main text, or Methods section.

n/a	Confirmed	
☐	☒	The exact sample size (*n*) for each experimental group/condition, given as a discrete number and unit of measurement
☒	☐	A statement on whether measurements were taken from distinct samples or whether the same sample was measured repeatedly
☐	☒	The statistical test(s) used AND whether they are one- or two-sided<br>*Only common tests should be described solely by name; describe more complex techniques in the Methods section.*
☐	☒	A description of all covariates tested
☐	☒	A description of any assumptions or corrections, such as tests of normality and adjustment for multiple comparisons
☐	☒	A full description of the statistical parameters including central tendency (e.g. means) or other basic estimates (e.g. regression coefficient) AND variation (e.g. standard deviation) or associated estimates of uncertainty (e.g. confidence intervals)
☐	☒	For null hypothesis testing, the test statistic (e.g. *F*, *t*, *r*) with confidence intervals, effect sizes, degrees of freedom and *P* value noted<br>*Give P values as exact values whenever suitable.*
☒	☐	For Bayesian analysis, information on the choice of priors and Markov chain Monte Carlo settings
☒	☐	For hierarchical and complex designs, identification of the appropriate level for tests and full reporting of outcomes
☐	☒	Estimates of effect sizes (e.g. Cohen's *d*, Pearson's *r*), indicating how they were calculated

Our web collection on statistics for biologists contains articles on many of the points above.

Software and code

Policy information about availability of computer code

Data collection	All data collection was performed manually
Data analysis	We have not developed any custom algorithms and only relied on publicly available softwares for sequencing analysis. In the Methods section of our manuscript we have provided details of the softwares, versions and parameters used for analysis.<br><br>List of bioinformatics packages used and version number where appropriate.<br>Trim Galore v0.6.6<br>Cutadapt<br>FastQC<br>Burrows-Wheeler Aligner (BWA) mem v0.7.17<br>Picard Tools v2.23.8<br>GATK v4.1.9.0<br>NGSCheckMate (downloaded March 15th 2018, commit number 1079908)<br>MuSE<br>LoFreq<br>Manta<br>Strelka<br>PureCN v1.16.0<br>MutationalPatterns

CNVkit v0.9.9
PyClone v0.13.1
ClonEvol (Version 0.99.11)
MultiQC (v1.9)
STAR 2.7.6a

QuantStudio6-Flex Applied Biosystems was used to perform RTqPCR assay, and for data analysis Design & Analysis software 2.6.0 was used.

For statistical analysis GraphPad Prism 9 or R statistical environment (v3.6.0) were used. Statistical overrepresentation test was carried out using the GO Biological Process (complete) annotation set of PANTHER database (http://www.pantherdb.org, analysis performed 12th July 2022).

Living image software (PerkinElmer, 4.7.3) was used for IVIS imaging analysis; NDP.view2 (2.7.52) was used for image viewing. EVOS M7000 software was used for acquisition of bright field and fluorescence images in vitro.

For manuscripts utilizing custom algorithms or software that are central to the research but not yet described in published literature, software must be made available to editors and reviewers. We strongly encourage code deposition in a community repository (e.g. GitHub). See the Nature Portfolio guidelines for submitting code & software for further information.

Data

Policy information about availability of data

All manuscripts must include a data availability statement. This statement should provide the following information, where applicable:

- Accession codes, unique identifiers, or web links for publicly available datasets
- A description of any restrictions on data availability
- For clinical datasets or third party data, please ensure that the statement adheres to our policy

Whole exome sequencing data was generated by Illumina NovaSeq 6000, RNASeq by llumina NovaSeq 6000 and Illumina HiSeq, and reads were aligned against the human assembly build GRCh38.

Data availability statement: Raw data from whole exome sequencing have not been deposited due to ethical considerations, however, supplementary tables listing the processed data for (a) somatic variants identified, and (b) CNA standardised Log2 ratios for all samples are provided. The RNAseq data is deposited in Sequence Read Archive (SRA) - ID number, PRJNA988939 https://www.ncbi.nlm.nih.gov/bioproject/?term=PRJNA988939. All other data generated in this study are provided in the Supplementary Information and Source Data files which accompany this manuscript. Correspondence and requests for further data or materials should be addressed to the corresponding author.

Research involving human participants, their data, or biological material

Policy information about studies with human participants or human data. See also policy information about sex, gender (identity/presentation), and sexual orientation and race, ethnicity and racism.

Reporting on sex and gender	All clinical material collected for this research was from female patients. Sample collection was permitted from male patients, however no male cases of breast cancer leptomeningeal metastasis (BCLM) were encountered during the time period of sample collection.
Reporting on race, ethnicity, or other socially relevant groupings	No socially relevant categorizations were used in this study
Population characteristics	Detailed clinical data for each patient is presented in Table 1, and group characteristics in Supplementary Table 1.
Recruitment	The individuals whose samples are included in this manuscript were identified by clinicians treating breast cancer, when those individuals were planned to undergo routine clinical testing for a diagnosis of leptomeningeal metastasis. Once identified, individuals gave their informed consent to the relevant clinical trial protocol listed, permitting collection of research samples. No self-selection biases have been identified.
Ethics oversight	All 21 patients in this study participated in NHS Health Research Authority (HRA) Research Ethics Committee (REC) approved studies (REC IDs 13/LO/1248 - South East London Cancer Research Network, UK and 14/LO/0292 - The Royal Marsden NHS Foundation Trust, UK).

Note that full information on the approval of the study protocol must also be provided in the manuscript.

Field-specific reporting

Please select the one below that is the best fit for your research. If you are not sure, read the appropriate sections before making your selection.

☒ Life sciences ☐ Behavioural & social sciences ☐ Ecological, evolutionary & environmental sciences

For a reference copy of the document with all sections, see nature.com/documents/nr-reporting-summary-flat.pdf

Life sciences study design

All studies must disclose on these points even when the disclosure is negative.

Sample size	For human material at the time of writing this manuscript, 58 samples from 21 patients were available for analysis. Sample size was based solely on availability of material and was not pre-determined. An overview of the samples collected is shown in Figure 1A. The number of patient derived organoid (PDO) models were 5. For PDO xenograft models, sample sizes were between 4 and 8 mice chosen to explore the variability in engraftment rates and metastatic phenotypes. No power calculation was used since these experiments were for model development rather than comparative evaluation.
Data exclusions	There were no data exclusions for human material studies. For xenograft mammary fat pad (mfp) models: mice which did not develop primary tumour or regressed in a cohort were excluded with exception of KCL320 in which data from all mice are shown. For intracardiac inoculation models mice with unsuccessful inoculations, as determined by IVIS immediately post inoculation, were excluded.
Replication	Sequencing analyses were undertaken de novo by an independent bioinformatician to ensure reproducibility of the findings. Immunohistochemical staining of PDOs was performed on 2 - 4 biological replicates (different passages of PDO) and all attempts were successful. In vivo experiments as shown in the manuscript were not replicated due to adherence to principles of 3 R's, however PDOs have been used in vivo for other projects in our laboratory, revealing similar growth rates and metastatic phenotypes.
Randomization	No randomisation was performed in this study since animal studies were for model development rather than comparative evaluation.
Blinding	Blinding was not required since this study was non-interventional.

Reporting for specific materials, systems and methods

We require information from authors about some types of materials, experimental systems and methods used in many studies. Here, indicate whether each material, system or method listed is relevant to your study. If you are not sure if a list item applies to your research, read the appropriate section before selecting a response.

Materials & experimental systems

n/a	Involved in the study	
☐	☒	Antibodies
☐	☒	Eukaryotic cell lines
☒	☐	Palaeontology and archaeology
☐	☒	Animals and other organisms
☐	☒	Clinical data
☒	☐	Dual use research of concern
☒	☐	Plants

Methods

n/a	Involved in the study	
☒	☐	ChIP-seq
☒	☐	Flow cytometry
☒	☐	MRI-based neuroimaging

Antibodies

Antibodies used	Alpha-catenin, Rabbit. Abcam (Cat No 51032), 1:100<br>Beta-catenin, Mouse. BD Biosciences (Cat No 610153), 1:2500<br>E-cadherin, Mouse. Dako (Cat No M3612), 1:50<br>ERα, Rabbit. Dako (Cat No M3643), 1:80<br>HER2 (HercepTest™), Dako. (Cat No SK001), N/A<br>Lamin A/C, Rabbit. Abcam (Cat No 108595), 1:750<br>p120-catenin, Mouse. BD Biosciences (Cat No 610134), 1:500
Validation	All antibodies used for IHC in this study are commercially available and have been validated by the manufacturer. However, to perform optimisation in our laboratory the following were used:<br>ER - A multipart dynamic range control consisting of breast cancer tissue previously reported as ER negative, ER strong and ER low expression in the diagnostic setting.<br>Lamin A/C and E-cadherin: normal human tonsil tissue was used.<br>HER2- A dynamic range control supplied with the clinical diagnostic kit was used according to manufacturer protocol.<br>Alpha-catenin- was tested on normal breast, DCIS, tonsil, liver (human) and liver (mouse).<br>Beta-catenin- was tested on tonsil and colon (human) as well as normal breast, lobular and ductal breast cancer.<br>p120-catenin- was tested on tonsil, normal breast and lobular and ductal breast cancer.

Eukaryotic cell lines

Policy information about cell lines and Sex and Gender in Research

Cell line source(s)	DU4475, MDA-MB-134-VI, T47D and SUM44PE cell lines were Isacke laboratory stocks. Patient derived organoids were derived in this study from female participants. MCF7 isogenic cell line pair was obtained from Professor Chris Lord (Institute of Cancer Research, UK), as published in Bajrami et al, Cancer Discovery 2018.
Authentication	All cell lines and patient derived organoids were authenticated by short tandem repeat testing using the GenePrint 10 ID System (Promega). For patient derived organoids, the patients germline sample was used for reference. No misidentified cell line was used.<br><br>For authentication, MDA-MB-134-VI, T47D, and SUM44PE were performed on 2021/01/19. DU4475 STR testing was performed on 2021/03/30. MCF7CDH1+/+ and MCF7CDH1-/- were performed on 2017/03/09.<br>KCL320 was tested on 2021-01-19, KCL 450, KCL566, KCL 622 and KCL625 were tested on 2021-03-30.<br><br>The results for all cell lines used agreed with the public databases, confirming the correct identity of each cell line used. The results for PDOs matched the original authentication and confirming the correct identity.
Mycoplasma contamination	All cell lines and patient derived organoids were tested regularly for mycoplasma contamination (MycoAlert; Lonza)
Commonly misidentified lines (See ICLAC register)	No commonly misidentified cell lines were used in this study.

Animals and other research organisms

Policy information about studies involving animals; ARRIVE guidelines recommended for reporting animal research, and Sex and Gender in Research

Laboratory animals	All experiment were performed with female 6-8 week NOD.Cg-Prkdcscid Il2rgtm1Wjl/SzJ (NSG) mice. All mice were housed in individually ventilated cages and kept at 21C ± 2C, humidity level between 45-65% and light/dark cycle of 12 hours. Mice were monitored daily by ICR Biological Services Unit staff and had food and water ad libitum.
Wild animals	No wild animals were used in this study.
Reporting on sex	Breast cancer predominately occurs in females (99% of cases) therefore animals models used were female.
Field-collected samples	No field-collected samples were used.
Ethics oversight	All animal work was carried out under UK Home Office Project Licenses 70/7413, P6AB1448A and PP4856884 granted under the Animals (Scientific Procedures) Act 1986 (Establishment Licence, X702B0E74 70/2902) and was approved by the "Animal Welfare and Ethical Review Body" at The Institute of Cancer Research (ICR).

Note that full information on the approval of the study protocol must also be provided in the manuscript.

Clinical data

Policy information about clinical studies

All manuscripts should comply with the ICMJEguidelines for publication of clinical research and a completedCONSORT checklist must be included with all submissions.

Clinical trial registration	REC ID 13/LO/1248, South East London Cancer Research Network, UK, and REC ID 14/LO/0292, Royal Marsden NHS Foundation Trust, UK
Study protocol	https://www.hra.nhs.uk/planning-and-improving-research/application-summaries/research-summaries/immunopathogenesis-and-molecularbiology-in-breast-cancer-subtypes/<br><br>https://www.hra.nhs.uk/planning-and-improving-research/application-summaries/research-summaries/abc-bio-biological-sample-collection-protocol/
Data collection	Samples were collected within the South East London Cancer Research Network, UK and Royal Marsden NHS Foundation Trust, UK between the time periods 16/12/2015 and 22/03/2019
Outcomes	Both clinical trials are biological sample collection studies therefore no primary or secondary outcome measures have been determined.
